# Observations on quality of life and disease control in patients with hereditary angioedema

**DOI:** 10.1093/skinhd/vzaf055

**Published:** 2025-07-25

**Authors:** Thomas Buttgereit, Annika Gutsche, Markus Magerl

**Affiliations:** Institute of Allergology, Charité–Universitätsmedizin Berlin, Corporate Member of Freie Universität Berlin and Humboldt-Universität zu Berlin, Berlin, Germany; Immunology and Allergology, Fraunhofer Institute for Translational Medicine and Pharmacology ITMP, Berlin, Germany; Institute of Allergology, Charité–Universitätsmedizin Berlin, Corporate Member of Freie Universität Berlin and Humboldt-Universität zu Berlin, Berlin, Germany; Immunology and Allergology, Fraunhofer Institute for Translational Medicine and Pharmacology ITMP, Berlin, Germany; Institute of Allergology, Charité–Universitätsmedizin Berlin, Corporate Member of Freie Universität Berlin and Humboldt-Universität zu Berlin, Berlin, Germany; Immunology and Allergology, Fraunhofer Institute for Translational Medicine and Pharmacology ITMP, Berlin, Germany

## Abstract

**Background:**

Lanadelumab is administered subcutaneously at 2- to 4-week intervals for long-term prophylaxis in hereditary angioedema (HAE). The effect of longer treatment intervals on disease control or disease-related quality of life (QoL) remains elusive.

**Objectives:**

To assess whether the reduction of the burden of treatment improves QoL.

**Methods:**

The treatment outcome of 56 patients with HAE treated with lanadelumab was assessed. The last documented treatment interval was extracted from patient records; individual disease control and QoL were assessed routinely with the Angioedema Control Test (AECT) and Angioedema Quality of Life Questionnaire (AE-QoL). The evaluation was based on the duration of the injection interval, i.e. equal to or less than 28 days (L ≤ 28) or more than 28 days (L > 28).

**Results:**

In total, 56 patients were included, 23 patients with L ≤ 28 and 33 with L > 28. In the L ≤ 28 group, the mean AECT was 14 points and the AE-QoL was 24 points. In the L > 28 group, the mean AECT was 15.5 points and the AE-QoL was 11.6 points.

**Conclusions:**

We conclude that lanadelumab injection intervals can be generously individualized in many patients with HAE while fully maintaining disease control and QoL.

## Teaser

The effect of longer treatment intervals of lanadelumab on disease control or disease-related quality of life (QoL) in hereditary angioedema (HAE) patients remains elusive. We evaluated the Angioedema Control Test and Angioedema Quality of Life questionnaire in HAE patients treated with lanadelumab injection intervals of 28 days or less and at intervals longer than 28 days. We found no impairment of disease control or QoL in patients treated with lanadelumab intervals longer than 4 weeks.

What is already known about this topic?

- Lanadelumab is an effective treatment in patients with hereditary angioedema (HAE).
- Although the recommended dosing interval is 2 or 4 weeks, other intervals, including intervals of more than 4 weeks, are often used in patients with HAE.

What does this study add?

- The optimal dosing interval must be assessed individually in all patients.
- Lanadelumab dosing intervals of >4 weeks do not negatively affect efficacy, disease control or disease-related quality of life in most patients.
- Intervals of >4 weeks can even have a positive effect on the disease-related quality of life, and reduce the costs in any case.

Hereditary angioedema (HAE) due to C1 inhibitor deficiency (HAE-C1INH) is a rare disease characterized by recurrent swellings. The ultimate treatment goals are ‘total disease control’ and ‘normalization of the patient’s life’, which can only be achieved with long-term prophylaxis (LTP).^1^ The currently approved first-line options for LTP are plasma-­derived C1 inhibitor (intravenous/subcutaneous), the oral kallikrein-inhibitor berotralstat, the monoclonal antibody against plasma-kallikrein lanadelumab (subcutaneous) and the monoclonal antibody against activated factor XII (FXIIa) garadacimab (subcutaneous).

The recommended dose of lanadelumab for patients aged 12 years and older is 300 mg at 2-week intervals. This interval can be extended to 4 weeks if patients are stable attack-free during treatment, particularly in patients with low bodyweight (Europe) or in patients who have been well-­controlled for 6 months (USA). At the Berlin Angioedema Center of Reference and Excellence (ACARE^2^), we extended the injection intervals by 3 days early on during treatment, in patients with complete response, regardless of bodyweight, until the appearance of prodromal signs or a breakthrough attack. Patients with prodromal signs and/or breakthrough attacks continued their treatment at a fixed interval that was two extension steps shorter.^3^ This procedure provides patients with a highly individualized treatment scheme, which is intended to guarantee freedom from symptoms with the longest possible injection interval and leads to high treatment satisfaction as well as a reduction in treatment costs.^4^ More importantly, the reduction of the burden of the treatment may come with improved disease-related quality of life (QoL).

## Patients and methods

We analysed 56 patients with HAE-C1INH who underwent the systematic interval extension procedure with lanadelumab described above at our ACARE.^3^ We extracted the last documented treatment interval from the patient records as well as levels of disease control and QoL, as assessed with the Angioedema Control Test (AECT) and the Angioedema Quality of Life questionnaire (AE-QoL), respectively. The AECT is a validated, retrospective four-item questionnaire to assess angioedema disease control in the previous weeks with a minimum of 0 points (no disease control) and a maximum score of 16 points (complete disease control). The cut-off value of the AECT for controlled disease is 10 points; the minimal clinically important difference (MCID) is 3 points.^5,6^

The AE-QoL is a validated questionnaire to assess QoL in patients with angioedema in the past weeks and consists of 17 questions from the domains functioning, fatigue/mood, fears/shame and nutrition. Lower AE-QoL scores signify lower QoL impairment; the MCID is 6 points.^7,8^

We compared disease control and the QoL of patients with a treatment interval of 28 days or less (L ≤ 28, i.e. 14–28 days, only one partially treatment-refractory patient in this group used an interval of 13 days) with those patients with an interval of more than 28 days (L > 28). Patients who used lanadelumab once a month were assigned an interval of 30 days.

## Results

**Figure 1:**
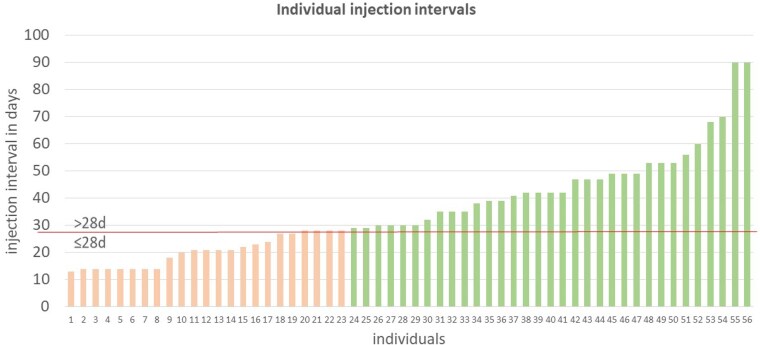
Injection intervals of lanadelumab in the presented patient cohort. The red line indicates the threshold between recommended dosing (up to 28 days, group L ≤ 28) and individual longer intervals (more than 28 days, group L > 28).

**Figure 2:**
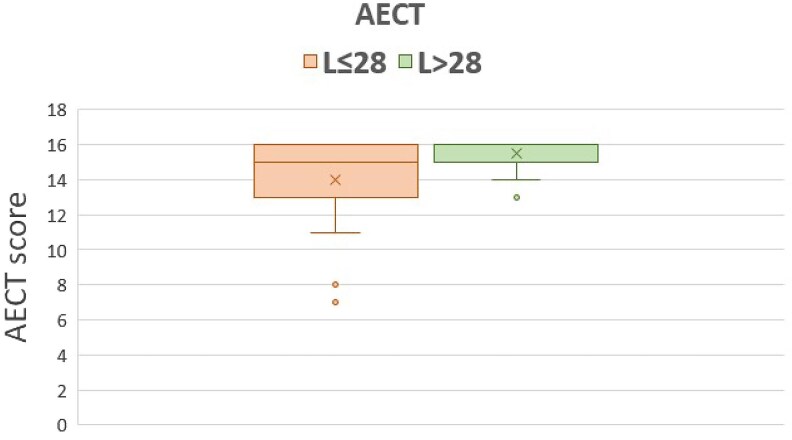
Angioedema Control Test (AECT) results in patients with hereditary angioedema due to C1 inhibitor deficiency using lanadelumab as long-term prophylaxis with injection intervals of 28 days or shorter (orange) and injection intervals of 29 days or longer (green). Boxplot: box, 25th to 75th percentile; horizontal line, median; cross, mean.

**Figure 3:**
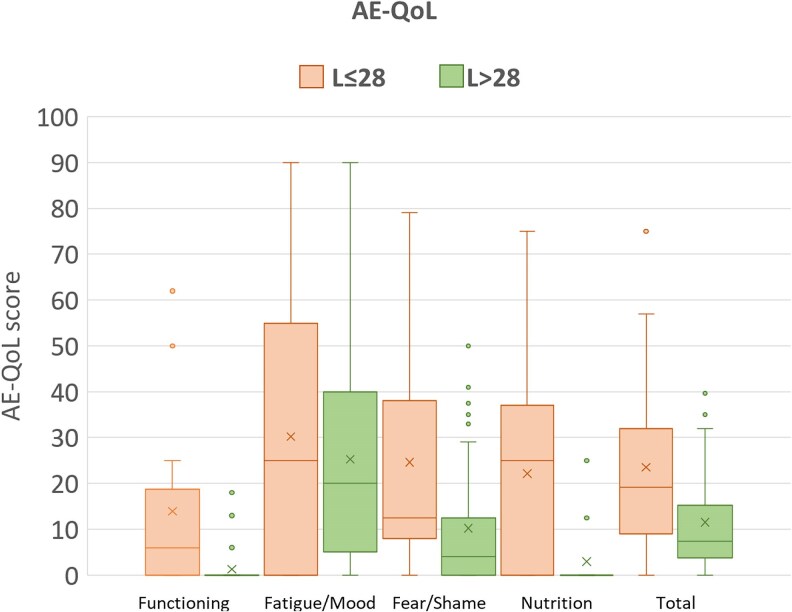
Disease-related quality of life (QoL) assessed by the Angioedema Quality of Life Questionnaire (AE-QoL) in patients with hereditary angioedema due to C1 inhibitor deficiency using lanadelumab as long-term prophylaxis with injection intervals of 28 days or shorter (L ≤ 28, orange bar of each couple) and injection intervals of 29 days or longer (L > 28, green bar of each couple). Boxplot: box, 25th to 75th percentile; horizontal line, median; cross, mean.

Our 56 patients used lanadelumab at a median treatment interval of 30 days (range 13–90), the mean interval was 35.5 days (SD 18) (Figure 1). Of these, 23 (70% female, *n* = 16) were L ≤ 28 patients; the L > 28 group consisted of 33 patients (64% female, *n* = 21). Before initiating lanadelumab, the mean AECT and AE-QoL scores between the L ≤ 28 and L > 28 groups did not significantly differ: 7.76 (SD 3.8) vs. 7.79 (SD 3.9) points for AECT, and 42.65 (SD 21.9) vs. 43.0 (SD 20.3) points for AE-QoL, respectively. During treatment with lanadelumab, the mean AECT score in the L ≤ 28 group was 14 points (SD 2.63), the AE-QoL score was 24 points (SD 19.5) (Figures 2, 3), and the domain scores for functioning, fatigue/mood, fears/shame and nutrition were 14, 30, 25 and 22 points, respectively. In the L > 28 group, the mean AECT score was 15.5 points (SD 0.9), the AE-QoL total score was 11.6 points (SD 10.7), and the domain scores for functioning, fatigue/mood, fears/shame and nutrition were 1, 25, 10 and 3, respectively (Figures 2, 3).

In the L ≤ 28 group, 43% of patients (*n* = 10) had complete disease control, while two patients had poorly controlled disease. The number of patients with AECT = 16 was significantly higher (exploratory *P* < 0.05, χ^2^ test) in the L > 28 group: 70% (*n* = 23) had complete control, and no patient had poorly controlled disease. The average AECT scores between L ≤ 28 and L > 28 patients were statistically different (exploratory *P* = 0.015, Mann–Whitney *U* test), but this difference (1.5 points) was lower than the MCID.^5,6^ The mean difference in the AE-QoL total scores of L ≤ 28 and L > 28 patients, 12 points, was statistically significant (exploratory *P* = 0.016, Mann–Whitney *U* test) and clinically meaningful (MCID = 6).

## Discussion

We show that patients with HAE-C1INH using lanadelumab at intervals of more than 28 days (off-label) have no loss of disease control or QoL, as compared with patients with shorter (in-label) intervals. The data suggest that patients with L > 28 even have an advantage in terms of a better QoL, particularly affecting the domains functioning, fears/shame and nutrition. A possible explanation for this could be the lower burden of treatment for patients with longer injection intervals (median: L ≤ 28 = 21 days vs. L > 28 = 42 days). Recently, Teixeira *et al*. reported that spacing dose administrations beyond 4-week intervals with maintained efficacy can increase patient convenience and concluded that injection intervals of lanadelumab exceeding 4-week can be considered on an individual basis.^9^

The burden of regular subcutaneous injections is often trivialized and held to be limited to the short-lived pain of injection. Our results suggest that this is not warranted and that a reduction of treatment applications may promote the normalization of life. The longer the treatment intervals, the less often patients have to think about their illness, which in our experience can even lead to patients forgetting their next injection or to carry on-demand treatment with them. Although this is problematic, it suggests that these patients experience a more normal life than patients who need to think about their treatment, and therefore their disease, more often.

Interestingly, patients with longer and shorter treatment intervals had well-controlled disease, on average, and the difference in disease control was not of clinical significance. This suggests that the burden of treatment may play a bigger role in QoL impairment rather than the burden that comes with having uncontrolled disease. Appropriate instruments and tools to measure specifically treatment burden should be used in the future to reinforce this observation. Limitations of the study are the observational and retrospective design. It could be assumed that patients who were able to successfully extend their lanadelumab treatment intervals (L > 28) and achieved better QoL did so because their disease was less severe. However, our data argue against this bias as L > 28 patients and L ≤ 28 patients did not differ in their AECT and AE-QoL scores before the initiation of lanadelumab. We cannot exclude that other factors may have influenced the ability to extend intervals. Our observations described here do not prove causality between the improved outcome and the extension of injection intervals.

Our real-world findings suggest that lanadelumab administration intervals may be extended in selected patients without compromising disease control. Prospective, controlled studies are warranted to confirm these observations, but the high degree of individualization in our approach could hardly be translated into a study protocol. Considering the monocentric design, the study included a relatively high number of patients with the rare disease HAE, but this is not yet sufficient to form further subgroups and gain deeper insights into the correlations.

In summary, individualizing the application intervals for HAE patients using subcutaneous LTP can optimize outcomes. In addition to reducing the need for treatment and costs, the QoL and disease control seem not to be impaired.

Please note, after data extraction, the partially responding patient (patient no. 1 in Figure 1) using a 13-day interval was switched to a daily dose of berotralstat 150 mg. He is now symptom-free.

## Data Availability

The data underlying this article will be shared on reasonable request to the corresponding author.
